# Novel chloroacetamido compound CWR-J02 is an anti-inflammatory glutaredoxin-1 inhibitor

**DOI:** 10.1371/journal.pone.0187991

**Published:** 2017-11-20

**Authors:** Olga Gorelenkova Miller, Kyle S. Cole, Corey C. Emerson, Dharmaraja Allimuthu, Marcin Golczak, Phoebe L. Stewart, Eranthie Weerapana, Drew J. Adams, John J. Mieyal

**Affiliations:** 1 Department of Pharmacology, Case Western Reserve University, Cleveland, Ohio, United States of America; 2 Department of Chemistry, Boston College, Chestnut Hill, Massachusetts, United States of America; 3 Department of Genetics, Case Western Reserve University, Cleveland, Ohio, United States of America; 4 Cleveland Center for Membrane and Structural Biology, Case Western Reserve University, Cleveland, Ohio, United States of America; University of Nebraska-Lincoln, UNITED STATES

## Abstract

Glutaredoxin (Grx1) is a ubiquitously expressed thiol-disulfide oxidoreductase that specifically catalyzes reduction of S-glutathionylated substrates. Grx1 is known to be a key regulator of pro-inflammatory signaling, and Grx1 silencing inhibits inflammation in inflammatory disease models. Therefore, we anticipate that inhibition of Grx1 could be an anti-inflammatory therapeutic strategy. We used a rapid screening approach to test 504 novel electrophilic compounds for inhibition of Grx1, which has a highly reactive active-site cysteine residue (pKa 3.5). From this chemical library a chloroacetamido compound, CWR-J02, was identified as a potential lead compound to be characterized. CWR-J02 inhibited isolated Grx1 with an IC_50_ value of 32 μM in the presence of 1 mM glutathione. Mass spectrometric analysis documented preferential adduction of CWR-J02 to the active site Cys-22 of Grx1, and molecular dynamics simulation identified a potential non-covalent binding site. Treatment of the BV2 microglial cell line with CWR-J02 led to inhibition of intracellular Grx1 activity with an IC_50_ value (37 μM). CWR-J02 treatment decreased lipopolysaccharide-induced inflammatory gene transcription in the microglial cells in a parallel concentration-dependent manner, documenting the anti-inflammatory potential of CWR-J02. Exploiting the alkyne moiety of CWR-J02, we used click chemistry to link biotin azide to CWR-J02-adducted proteins, isolating them with streptavidin beads. Tandem mass spectrometric analysis identified many CWR-J02-reactive proteins, including Grx1 and several mediators of inflammatory activation. Taken together, these data identify CWR-J02 as an intracellularly effective Grx1 inhibitor that may elicit its anti-inflammatory action in a synergistic manner by also disabling other pro-inflammatory mediators. The CWR-J02 molecule provides a starting point for developing more selective Grx1 inhibitors and anti-inflammatory agents for therapeutic development.

## Introduction

Inflammation has long been recognized as a deleterious contributing factor in numerous disease conditions, prompting continuous pursuit of effective anti-inflammatory agents for therapy. In this context, many protein mediators of pro-inflammatory signaling are known to undergo reversible redox modifications on cysteine residues that can regulate their functions. Considering the intracellular abundance of GSH and the propensity of these modified cysteine residues to react with GSH, it is expected that a prevalent outcome of redox signaling is protein-S-glutathionylation (protein-SSG). Thus, regulation of reversible protein-SSG formation has become a central issue in inflammatory responses and neurodegenerative diseases [1, 2], focusing attention on the enzyme glutaredoxin (Grx1).

Grx1 is a ubiquitously expressed oxidoreductase that efficiently and specifically catalyzes deglutathionylation of mixed disulfide (S-glutathionylated) substrates [3]. Grx1 has been found to promote transcription of pro-inflammatory genes *via* deglutathionylation of members of the pro-inflammatory NFκB transcription pathway (reviewed in [1, 2]). Grx1 has been implicated as a positive regulator of inflammation in numerous contexts, such as diabetic retinopathy, cigarette smoke-induced inflammation and allergic airway response, and microglial activation [4–6]. Adenoviral overexpression of Grx1 alone; i.e., in the absence of pro-inflammatory stimuli, has been shown to increase release of pro-inflammatory markers from model retinal glial cells, epithelial cells, and microglial cells [4–6]. Moreover, Grx1 is upregulated by various inflammatory stimuli in peripheral immune and epithelial cells [7–9], and in microglia [6], thereby potentially creating a feed-forward loop of inflammatory propagation. Grx1 silencing inhibits pro-inflammatory cytokine release in both cell culture and animal models of inflammatory disease [4, 5, 10]. In addition, *Grx*^-/-^ mice demonstrate blunted cytokine release from airway epithelial cells in response to both cigarette smoke and ovalbumin challenge used to simulate allergic airway response [4, 11]. The accumulated evidence for a pro-inflammatory regulatory role of Grx1 suggests inhibition of Grx1 as a potential anti-inflammatory strategy. Therefore we set out to discover an inhibitor of Grx1 that was effective intracellularly in model microglial cells and test whether such an inhibitor would display anti-inflammatory efficacy.

To date, no selective inhibitors for Grx1 have been identified that are effective in cells. Grx1 cannot be inhibited by reversible binding of substrate analogs due to its high commitment to covalent catalysis [3]; i.e., the rates of the covalent reactions of the nucleophilic double displacement mechanism far exceed the rates of dissociation of each of the substrates, precluding competitive inhibition by substrate analogs. Thus, targeting the active site of Grx1 non-covalently is impractical. Therefore, in the current study we used a stochastic rapid screening approach and focused on thiol-selective electrophilic compounds that would be likely to covalently adduct the nucleophilic active site cysteine of Grx1. We adapted the standard spectrophotometric assay for Grx1 activity to test 504 novel thiol-reactive compounds. Of these, the methyl 3-((4-(2-chloroacetamido) benzyl) carbamoyl)-5-(prop-2-yn-1-yloxy) benzoate, “CWR-J02,” emerged as the most promising for validation as a Grx1 inhibitor. Hereafter the compound will be referred to as “J02.” J02 was found to inhibit isolated Grx1 in a concentration- and time-dependent fashion, with selectivity for Grx1 relative to glutathione disulfide reductase. With the BV2 microglial cell line, J02 was found to inhibit Grx1 activity without altering Grx1 content; and to decrease lipopolysaccharide (LPS)-stimulated cytokine gene expression, documenting anti-inflammatory activity. Selective knockdown of Grx1 in the microglia led to anti-inflammatory effects as expected, but treatment with J02 displayed greater anti-inflammatory impact, suggesting additional targets of J02 contributing to the overall effect. Indeed, affinity precipitation and mass spectrometric analysis of the J02-adducted proteome revealed numerous potential targets of J02. Notably, Grx1, and several components of the NFκB pro-inflammatory signaling pathway were detected; however, the extent of adduction and functional consequences are not detectable by this analysis. Taken together, these data suggest that J02 may serve as a Grx1 inhibitor and an anti-inflammatory agent *in vivo*, serving as a starting point for developing more effective Grx1 inhibitors, and/or anti-inflammatory agents for *in vivo* and therapeutic applications. This study identifies the first reported covalent modifier for Grx1 that is effective intracellulary, providing a lead compound that can then be further optimized for selectivity. Notably, the covalent mode of action and the presence of the alkyne group on the J02 molecule make it relatively facile to screen related derivatives for selectivity and intracellular potency in future studies.

## Results

### Chloroacetamido compound J02 inhibits the activity of Grx1 *via* covalent modification

**Fig 1:**
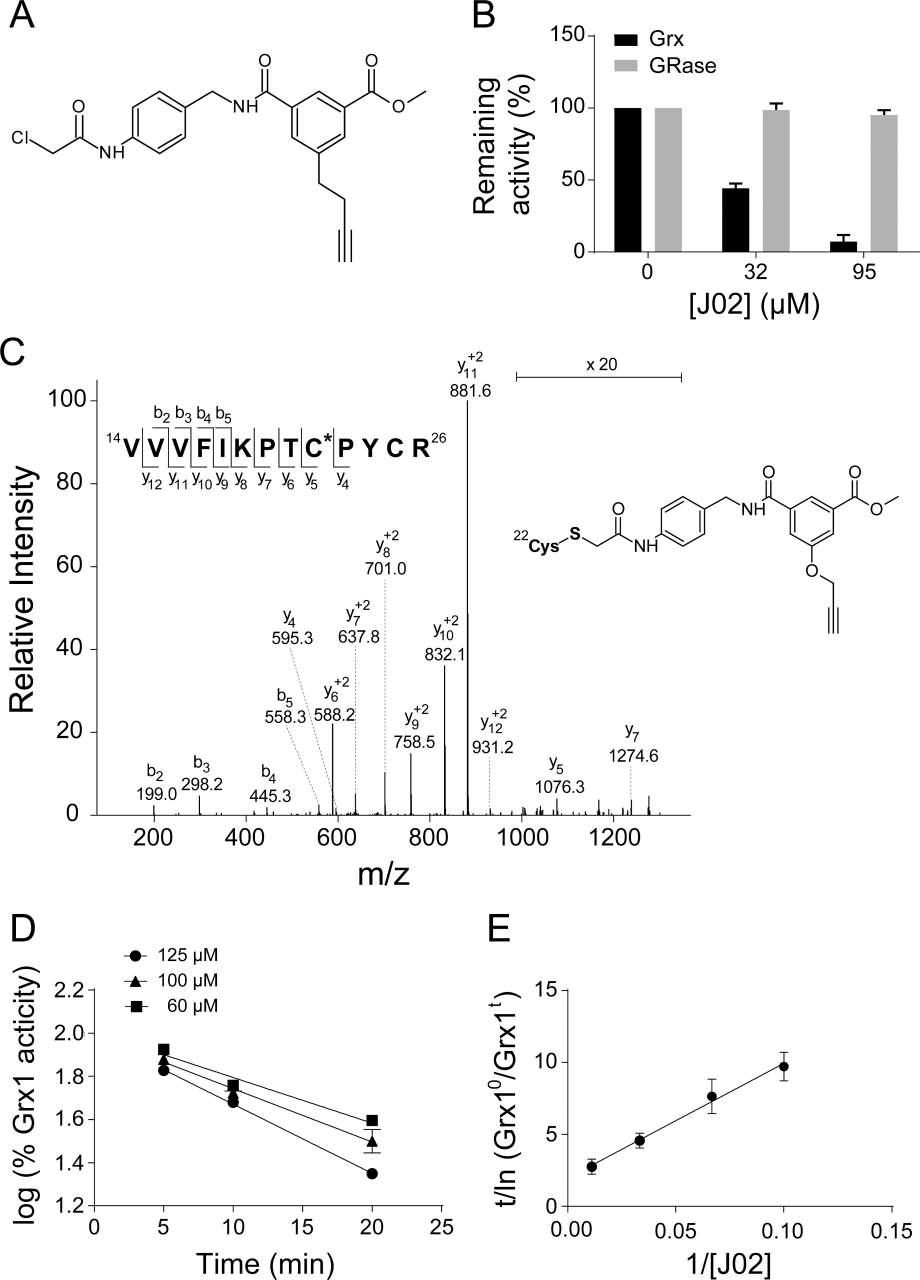
Novel chloroacetamide J02 inhibits Grx1 as isolated enzyme. **A**, J02 chemical structure. **B**, % enzyme inhibition by J02 of Grx1 or GR as isolated enzymes. Grx1 or GR were pre-incubated with indicated concentrations of J02 in complete assay mix for 30 min. Enzyme activity was then measured using standard spectrophotometric assays. n≥3±SEM. **C**, Identification of J02 adducted to the active site cysteine of Grx1 by mass spectrometry. The tandem spectrum was collected for the m/z 654.3 [M+H]^+3^ ion that corresponds to the peptide ^14^VVVFIKPTCPYCR^26^ modified by J02 adduction at Cys-22 and carbamidomethylation at Cys-25. Fragmentation of the parent ion revealed the presence of the cysteinyl-J02 moiety identified by a series of unique and subsequent “y” ions (y_4_ –y_12_). These fragments unambiguously confirm Cys-22 as the site of J02 adduction. The inset on the upper left of panel C of **Fig 1** indicates the observed fragment ions of the peptide containing modified Cys-22, labeled according to Biemann nomenclature. Of the five cysteine residues on Grx1, only cysteine-22 was found to be adducted under these conditions. **D**, J02 inhibition of Grx1 isolated enzyme activity in a concentration- and time-dependent manner. Grx1 (40 milliunits (nmol substrate/min)) was incubated with indicated concentrations of J02 in 0.33 M sodium potassium phosphate buffer pH 7.4 at 30°C for indicated time. The mixture was then diluted 20-fold into complete assay mix, and standard spectrophotometric assay was performed. **E**, modified Kitz-Wilson plot for Grx1 inactivation by J02. Grx1 (4 milliunits) was pre-incubated with various (10–90 μM) concentrations of J02 in 0.33 M sodium potassium phosphate buffer pH 7.4 for 5 min at 30°C. A separate experiment verified the log linear relationship between J02 concentration and % Grx1 inhibition for the range of experimental conditions (see **Fig 1D**). K_*I*_ and k_*inact*_ were determined according to the relationship ln(E_0_/E_t_)/t = k_i*nact*_[I]/(K_*I*_ + [I]), where E_0_ refers to Grx1 activity at time zero, and Et refers to Grx1 activity after 5 min pre-incubation, [I] refers to J02 concentration, K_*I*_ is the concentration of J02 that gives half the maximal rate of inactivation, and k_*inact*_ is the net rate constant for inactivation. The K_*I*_ for J02 is 40 μM and k_*inact*_ is 0.5 min^-1^. n = 3 ± SEM.

Chloroacetamides are known to be thiol-reactive, and Grx1 has an especially reactive, low pKa active site thiol, Cys-22 [12]. We hypothesized that compounds with the chloroacetamido moiety (i) would covalently adduct the Grx1 active site and inactivate the enzyme, and (ii) would be relatively selective for Grx1, reacting more rapidly with its Cys-22 moiety than with other protein-SH groups. Accordingly, we assayed a chemical library of 504 electrophilic compounds, most of which are thiol-selective, in a rapid screening fashion. Of these, compound J02 (**Fig 1A**) elicited the greatest decrease in Grx1 activity according to the standard assay adapted for rapid endpoint analysis of micro samples in a 384-well plate.

To characterize J02 as a *bona fide* Grx1 inhibitor, we investigated the concentration-dependent effects of J02 on the activity of the isolated enzyme. Pre-incubation of isolated Grx1 for 30 min with various concentrations of J02 in the presence of 1 mM GSH resulted in a concentration-dependent loss of activity, with IC_50_ = 32 μM (**Fig 1B**). In contrast, J02 did not affect the activity of glutathione disulfide reductase (GR) when tested separately under analogous conditions (**Fig 1B**). Importantly, this confirms that any inhibitory effects observed in the assay of Grx1 activity, which includes GR as the coupling enzyme, were not due to direct inhibition of GR.

#### Identification of site of J02 covalent modification of Grx1

Considering the chemical nature of J02, we expected that inactivation of Grx1 could be attributed to covalent adduction of J02 to the active site cysteine (Cys-22). This prediction was confirmed by tandem mass spectrometric analysis of the tryptic peptides derived from J02-treated Grx1 (**Fig 1C**). Grx1 sequence coverage included all cysteine residues present in the protein. Detailed analysis of MS data revealed the addition of de-chlorinated J02 (+378.2 Da) to a peptide containing the catalytic cysteine (^14^VVVFIKPTCPYCR^26^). The fragmentation pattern of this peptide identified the exclusive location of the covalent modification to be the side chain of Cys-22 (**Fig 1C**). Tandem mass spectrometry showed that fragmentation of the parent ion gave a fragment ion of m/z 654.3 [M+H]^+3^ that corresponds to the ^14^VVVFIKPTCPYCR^26^ peptide. Further fragmentation revealed the presence of the cysteinyl-J02 moiety identified by a series of unique and subsequent “y” ions (y_4_ –y_12_). These fragments unambiguously confirm Cys-22 as the site of J02 adduction. The inset on the upper left of panel C of **Fig 1**indicates the observed fragment ions of the peptide containing modified Cys-22, labeled according to Biemann nomenclature. Of the five cysteine residues on Grx1, only cysteine-22 was found to be adducted under these conditions.

Consistent with the covalent mechanism of inactivation of Grx1, pseudo first order kinetics was observed (**Fig 1D**) for the time-dependent and J02 concentration-dependent loss of Grx1 activity. When re-plotted in a modified Kitz and Wilson format [13] **(Fig 1E**), KI for J02 was found to be 40 μM, and k_*inact*_ was 0.5 min^-1^.

### Molecular dynamics simulation suggests non-covalent interactions of J02 on Grx1

**Fig 2:**
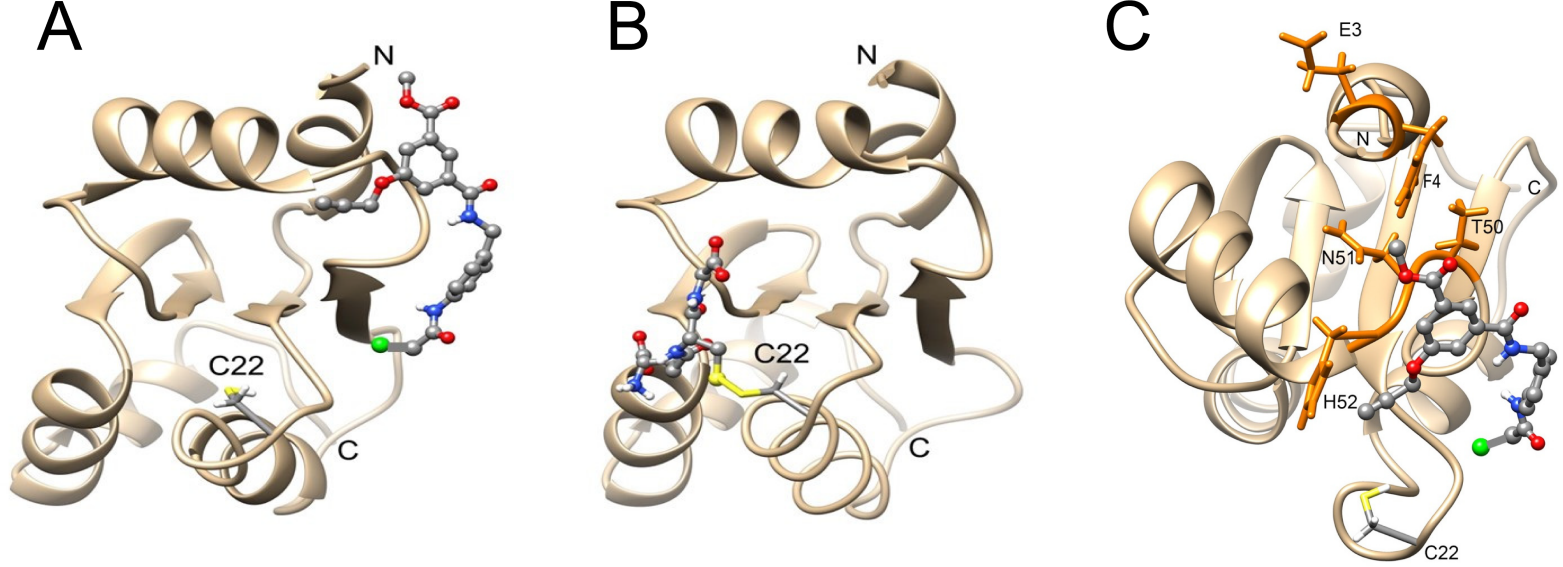
MD simulations suggest that J02 forms an interaction with Grx1 in a distinct region of Grx1 compared to that of the glutathionyl moiety in the Grx1 glutathionyl mixed disulfide intermediate. **A**, Grx1/J02 complex at the end of a 50 ns molecular dynamics simulation, using the NMR structure of Grx1 (PDB ID: 1JHB); the chlorine atom of J02 is shown in green. **B**, NMR structure of the glutathionyl mixed disulfide intermediate of the quadruple C—>S mutant of Grx1 (QM-Grx1-Cys22-SSG) (PDB ID: 1B4Q). Approximately the same view of Grx1 is shown in panels A and B. **C**, Rotated view of the Grx1/J02 complex, highlighting the main interaction residues of Grx1 in orange (Glu-3, Phe-4, Thr-50, Asn-51, and His-52). The catalytic Cys-22 is labeled in all three panels.

To gain insight regarding potential non-covalent interactions of J02 with Grx1, preceding the covalent adduction of Cys-22, we used a molecular dynamics approach to simulate the J02-Grx1 interaction for 50 ns, initially positioning the chlorine-bearing carbon of J02 in the vicinity of Cys-22 of the NMR structure of Grx1. The resulting complex after 50 ns shows several features. The methylene carbon of the chloroacetamido group of J02 was found to be within 10 Å of the sulfur atom of Cys-22. The rest of the J02 molecule was folded over in a relatively extended conformation, placing several of the oxygen and nitrogen atoms of J02 near Thr-50 and other neighboring residues capable of forming hydrogen bonds at a recognition site on Grx1 (**Fig 2A**). The binding site on Grx1 identified by the molecular dynamics (MD) simulation for the interaction with J02 is distinct from that involved in stabilizing the Grx1-SSG catalytic intermediate (**Fig 2B**). In the simulated complex, J02 interacts primarily with Grx1 residues Glu-3, Phe-4, Thr-50, Asn-51, and His-52 (**Fig 2C**). The prediction of an initial reversible binding site preceding covalent thioether formation with Cys-22 is consistent with the observed kinetics displayed in **Fig 1E**, where the non-zero *y*-intercept of the Kitz- Wilson plot indicates a reversible non-covalent association of J02 with Grx1 preceding covalent adduction. The finding *via* molecular dynamics simulation of a distinct binding site for J02 suggests that the area surrounding Thr-50 may represent a novel recognition site on Grx1 that could be exploited for further inhibitor development.

### J02 inactivates Grx1 in microglia

**Fig 3:**
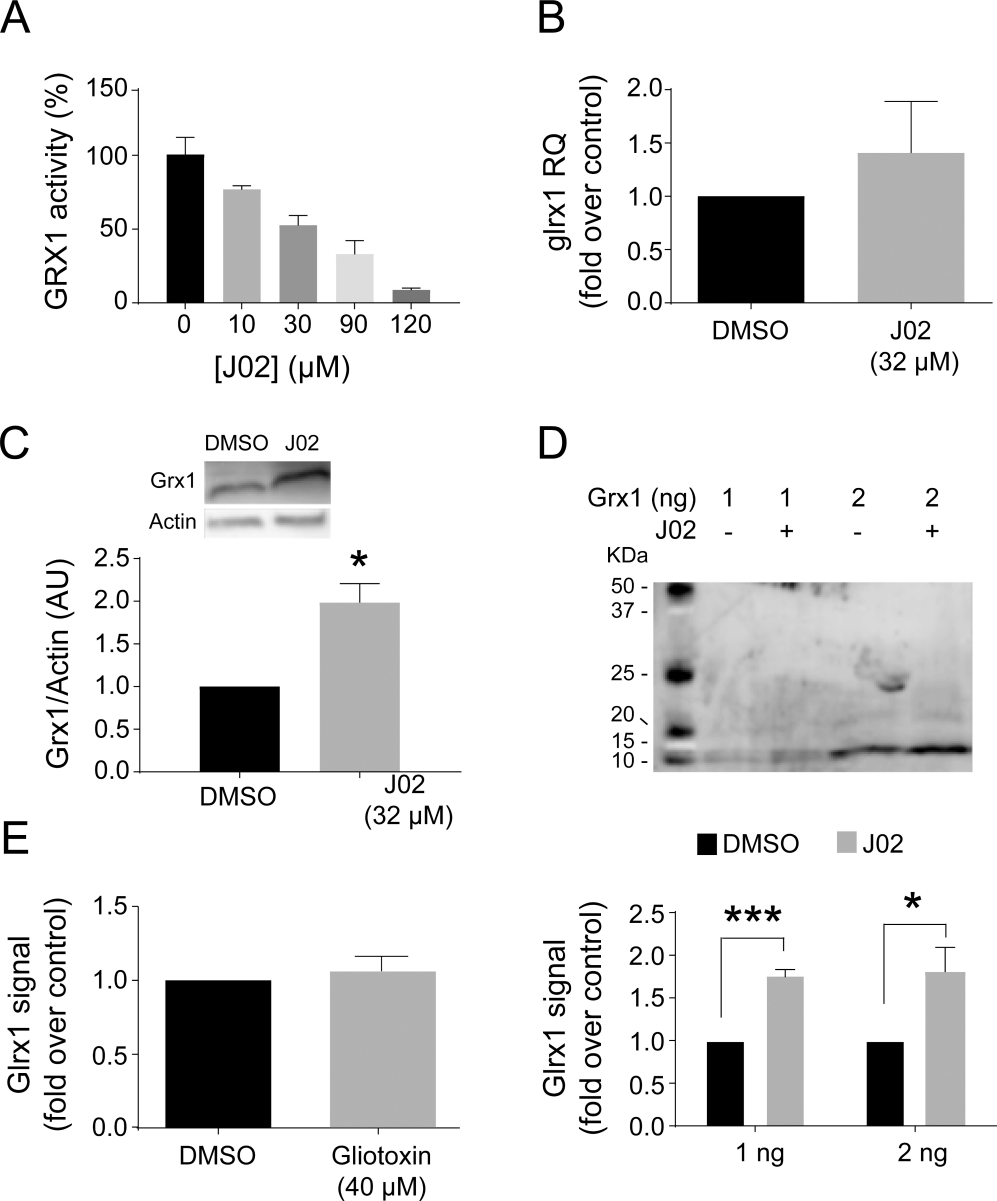
J02 inhibits Grx1 in BV2 murine microglia cells. **A**, Grx1 specific activity in BV2 cell lysates. Cells were treated with 32 μM J02 or DMSO for 30 min, medium changed, and cells were allowed to recover for 1 hour before lysing and assaying activity. **B**, *glrx1* mRNA levels in BV2 cells treated as in A, or BV2 cells pre-incubated with 32 μM J02 or DMSO for 30 min and then treated with 1 μg/ml LPS for 1 hour. **C**, Immunoblot of BV2 murine microglial cells treated as in **A**. Densitometric quantification on right. **D**, Immunoblot of purified Grx1 incubated with 96 μM J02 or DMSO. Incubation was performed in phosphate buffer pH 7.4 for 30 min at room temperature. Densitometric quantification is shown on right. E, Grx1 activity in BV2 cells treated with 40 μM gliotoxin (sporidesmin analog) or DMSO as in A. n = 3 ± SEM. *p<0.05, **p<0.01, ***p<0.001. RQ—relative quantity.

In order to examine the effects of J02 on Grx1 function in its natural environment, we treated intact cells with J02. We used the BV2 microglial cell line, which we previously characterized with regard to Grx1-dependent regulation of the inflammatory response [6]. Pre-treatment of the cells with various concentrations of J02 for 30 min resulted in a concentration-dependent loss of Grx1 activity, with an IC50 of 37 μM (**Fig 3A**), closely corresponding to the IC50 for the isolated enzyme that was preincubated for the same time period. This result documents the utility of the experimental design. Including 1 mM GSH in the reaction mixture for testing the inhibitory effect of J02 on the isolated Grx1 enzyme mimics the intracellular content of GSH, which is the major thiol-containing competitor for J02 on a mass-action basis. In contrast to Grx1, treatment of the BV2 microglia with 40μM J02 did not inhibit the activity of the related thiol-disulfide oxidoreductase enzyme thioredoxin (Trx1, supplemental **S3 Fig**), consistent with the much higher pKa values (~7–8) for the active site thiols of Trx1. Treatment of the cells with the IC50 concentration of J02 did not affect levels of *glrx1* mRNA, indicating that J02 did not interfere with Grx1 expression (**Fig 3B**). To further distinguish whether J02 decreased only the enzymatic function of Grx1 and not also its content, we measured the Grx1 content directly by Western blot (**Fig 3C**). To our surprise the Grx1 content appeared to increase after J02 treatment. However, this apparent change was documented to be an anomaly attributable to increased immunoreactivity of the J02-Grx1 adduct (**Fig 3D**), confirming that J02 treatment of cells did not lead to a change in Grx1 content.

We had previously characterized epidithiopiperazine-2,5-dione compounds (sporidesmin and gliotoxin) as inactivators of the isolated Grx1 enzyme [13]. However, these bridged disulfide-compounds were ineffective as Grx1 inhibitors in the presence of GSH. Accordingly, we expected that gliotoxin would not be effective as an inhibitor of Grx1 intracellularly where GSH is abundant. Indeed, treatment of microglial cells for 30 min with 40 μM gliotoxin had no effect on the activity of Grx1 (**Fig 3E**).

### J02 treatment of microglia is anti-inflammatory

**Fig 4:**
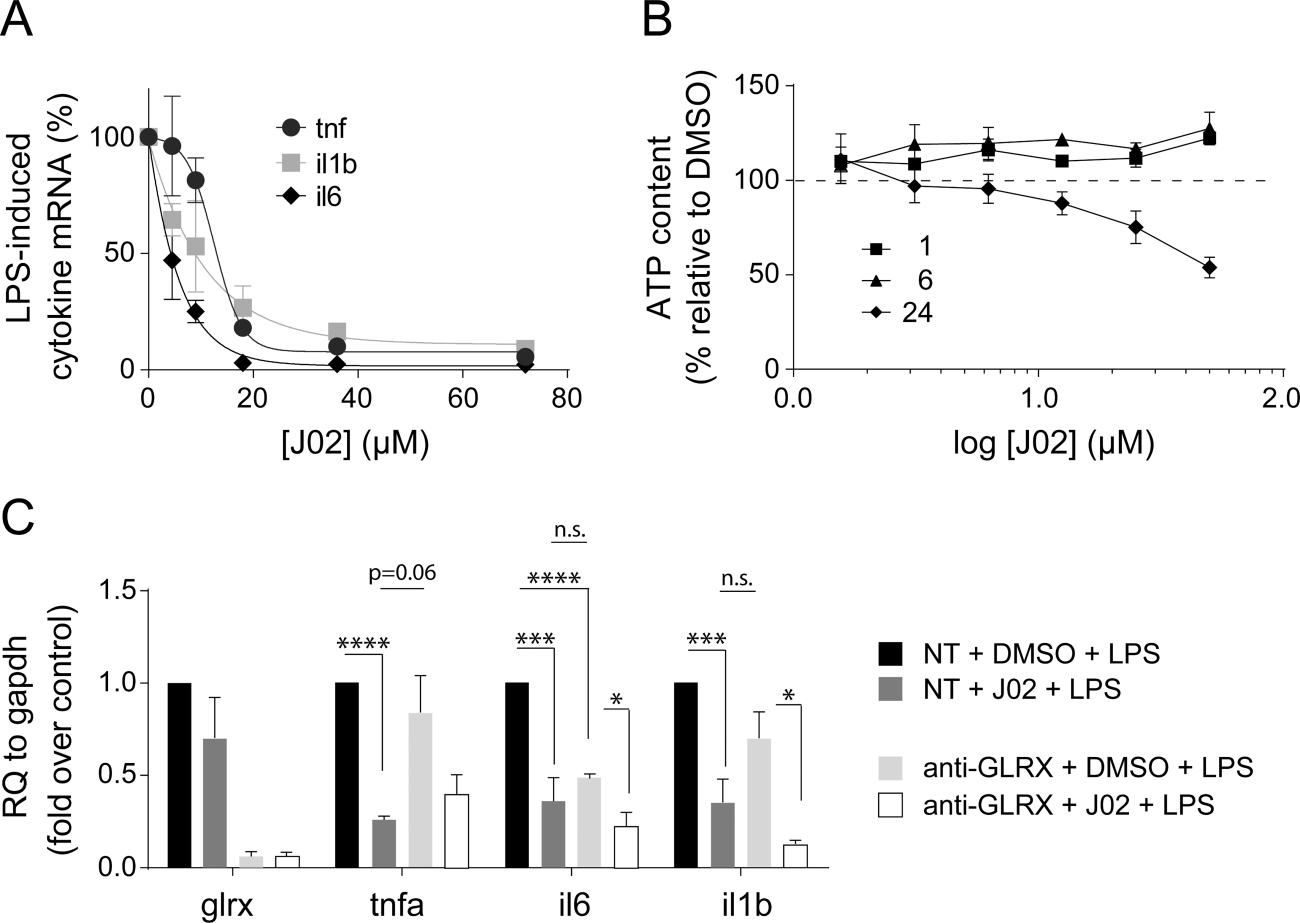
J02 inhibits cytokine expression in BV2 cells, not due to induction of cytotoxicity. **A**, Cytokine mRNA levels in lysates from BV2 cells pre-treated with indicated concentrations of J02 for 30 min and stimulated with 1μg/ml LPS for 60 min. n = 3 ± SEM. **p<0.01. RQ—relative quantity. **B**, ATP content in lysates from BV2 cells pre-treated with indicated concentrations of J02 for 30 min, allowed to recover for indicated amount of time. ATP levels were normalized to those from control (DMSO treated) cells. n = 3 ± SEM. Arrow indicates J02 concentration used in cell-based assays. **C**, mRNA levels in BV2 treated with non-targeting (NT) scrambled siRNA (control) or Grx1-targted (GLRX) siRNA for 24 hours, treated with J02 (32 μM) or DMSO for 30 min, and stimulated with 100 ng/ml LPS for 24 hours. n ≥ 3 ± SEM. *p<0.05, **p<0.01, ***p<0.001, ****p<0.0001. RQ—relative quantity.

We and others have observed previously that diminution of Grx1 content decreases cytokine expression in response to inflammatory stimulation in microglial cells [6], retinal glial cells [9], and alveolar macrophages [4], suggesting that a small molecule targeted to Grx1 may be anti-inflammatory. Accordingly, we investigated the effects of the Grx1 inhibitor J02 on cytokine expression in BV2 microglial cells. Pre-treatment of BV2 cells for 30 min with various concentrations of J02 decreased the LPS-induced levels of *tnfa*, interleukin-6 (*il6*), and interleukin-1 beta (*il1b*) in a concentration-dependent fashion (**Fig 4A**), consistent with a pronounced anti-inflammatory effect. We separately confirmed that the J02-dependent inhibition of LPS-induced cytokine expression was not due to a cytotoxic effect of J02. Thus, no ATP loss was observed up to 6 hours post 30 minute pretreatment with various concentrations of J02 (**Fig 4B**).

To assess the contribution of Grx1 deactivation toward the anti-inflammatory effects of J02, we selectively diminished Grx1 content in the BV2 cells *via* siRNA, as previously [[6]]. Grx1 knockdown to ~80% recapitulated the inhibitory effect of J02 on the LPS-induced elevation of *il6* and *il1b*, but not *tnfa* (**Fig 4C**). Grx1 knockdown, however, did not prevent J02-dependent inhibition of cytokine induction by LPS, suggesting that deactivation of Grx1 cannot be the only mechanism by which J02 exerts its anti-inflammatory effects. Therefore, we investigated the potential cellular targets of J02.

### J02 interactome in microglia

**Fig 5:**
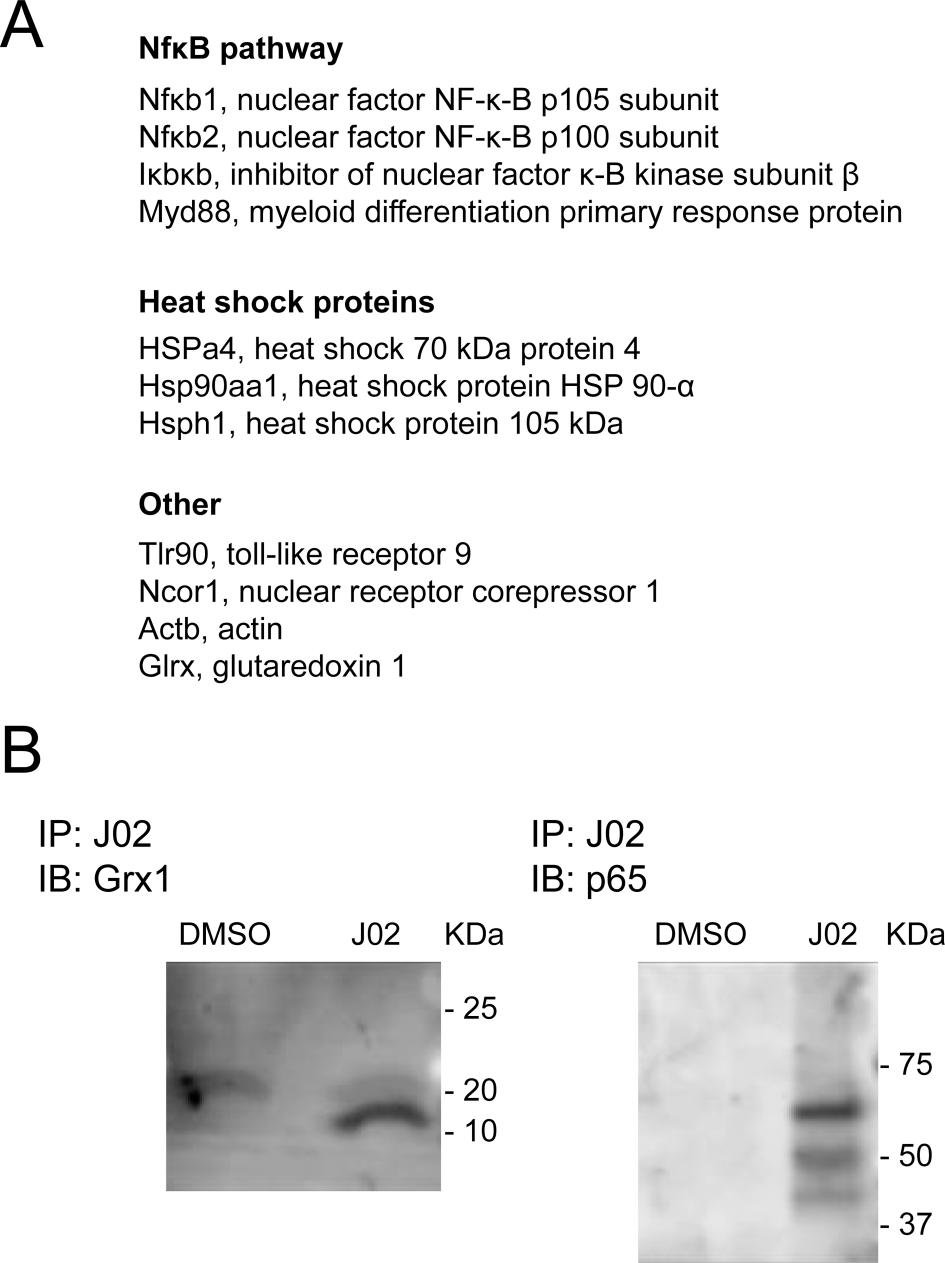
J02 interactome for BV2 cells–proteins involved in inflammatory responses. BV2 cells were treated with 40 μM J02 or DMSO. Resulting cell pellets were lysed, linked to a biotin azide probe, and run over a streptavidin column. **A**, Pulled down proteins were identified using mass spectrometry (see SI Materials and Methods section for further details). A, Inflammatory proteins shown to be regulated *via* S-glutathionylation and identified in the mass spectrometry dataset (supplemental **S1 Table**), including glutaredoxin-1. **B**, Grx1 and p65 are detected in J02-adducted samples. BV2 cells were treated with 40 μM J02 or equivalent volume DMSO for 30 min. Medium was changed, and cells were allowed to recover for 60 min. Resulting cell pellets were lysed, adducted with azide fluorescent fluorophore, and run over streptavidin beads to precipitate J02-adducted proteins. See Materials and Methods for further details. Eluted proteins were separated on SDS-PAGE gel, transferred to PVDF membrane, and probed with antibodies against Grx1 (left) and p65 (right).

The alkyne moiety on the J02 molecule facilitated a survey of intracellular targets of J02. Thus, using azido-substituted biotin and click chemistry, followed by pull down of J02-adducted proteins with streptavidin beads, the J02-targeted proteins were separated from J02-treated BV2 cell lysates. Then tryptic digests of the eluted proteins were analyzed by tandem mass spectrometry. In this way, numerous proteins were identified as being adducted to some extent by J02. Consistent with the anti-inflammatory efficacy of J02, Grx1 was identified among the proteins adducted by J02 in the microglial cells, as expected. In addition, a number of other pro-inflammatory proteins were identified by the mass spectrometric analysis (**Fig 5A**), including members of the NFκB pathway known to be regulated *via* S-glutathionylation. Two of these key proteins involved in regulation or mediation of NFκB signaling were further documented to be adducted by J02 by Western blotting with the corresponding antibodies for Grx1 and p65 after streptavidin pulldown (**Fig 5B**).

## Discussion

### Thiol-reactive compounds as anti-inflammatory agents

Covalent compounds targeted to cysteines have been shown to be anti-inflammatory in cell and animal models of disease. For example, inhibiting IL-2-inducible tyrosine kinase with a covalent inhibitor targeted towards the ATP binding site was found to decrease inflammation in human lung fragments [14]. The natural compound andrographolide was reported to exert anti-inflammatory action by covalently modifying Cys-62 of p50 [15]. 15d-PGJ_2_, a known anti-inflammatory prostaglandin, covalently binds to a number of cysteines [16, 17]. Thus, cysteine-targeted covalent modifiers have been found to be effective in decreasing inflammation.

We set out in the current study to test the hypothesis that inhibition of Grx1 would correspond to an anti-inflammatory effect. The thiol-reactive CWR-J02 compound does inhibit Grx1 intracellularly and it does elicit an anti-inflammatory effect; however the anti-inflammatory effect likely involves additional inflammatory mediator proteins as targets and it may also act on other side targets not involved in inflammation. These findings identify J02 as a valuable lead compound for further development with likely divergent goals. One goal would be to optimize the anti-inflammatory effect by characterizing the degree of inhibition of each of the other inflammatory protein targets of J02 and then modify its structure to enhance their inhibition while minimizing off-target effects. The other goal would be to seek more selective inhibitors of Grx1 by modifying the J02 structure, as considered next.

### J02 as a glutaredoxin inhibitor

J02 is a thiol-reactive agent based on its electrophilic chloroacetamido moiety, analogous to the simpler compound iodoacetamide; however J02 and related compounds with more complex structures have the potential for selectivity based on non-covalent interactions. A previously identified Grx1 inhibitor, sporidesmin, was characterized as a mechanism-based inhibitor that crosslinks two of the Grx1 cysteine residues [13]. However, the efficacy of sporidesmin as a Grx1 inhibitor in cells or *in vivo* was not explored. As GSH was shown to abrogate inhibitory action of sporidesmin *in vitro* due to reduction of the strained disulfide bond, it is likely that *in vivo*, where the intracellular content of GSH is relatively high, sporidesmin would not act as a Grx1 inhibitor. Our current data for the sporidesmin analog gliotoxin confirmed this supposition (**Fig 3E**). More recently, γ-glutamyldehydroalanyl-glycine, a metabolite of the DNA alkylating agent busulfan, was shown to covalently bind to, and inhibit, Grx1 and Grx2 *in vitro* [12]. Follow-up studies showed that busulan itself can inactivate Grx1 directly through a novel mechanism that crosslinking of Grx1 active site cysteine residues, similar to the inactivation mechanism employed by gliotoxin [13]. However, busulfan is a cytotoxic agent by design, and these studies did not include characterization of selectivity or relative potency for inactivation of isolated Grx or intracellular Grx [12]. In contrast, J02, even in the presence of 1 mM GSH, exhibits ~10-fold greater inhibitory potency toward isolated Grx1 (**Fig 1B)** compared to sporidesmin’s potency in the absence of GSH [13]. The K_*I*_ for J02 is 3-fold less than that of sporidesmin [13], again suggesting that J02 inhibits Grx1 at lower concentrations relative to sporidesmin; whereas the k_*inact*_ values for sporidesmin and J02 are similar (0.72 and 0.5 min^-1^, respectively). Previously, we found that CdCl_2_ is capable of inhibiting Grx1 in cells [18]; however, cadmium is a known health hazard, and it is not selective for Grx1. In addition, studies of Cd in the μM range generally have found it to be pro-inflammatory rather than anti-inflammatory [19].

In our current study, *in silico* modeling provided data that are predictive of hydrogen bonding between J02 and a recognition site on Grx1 (**Fig 2**). Such non-covalent interactions may confer selectivity for Grx1. Accordingly, the area surrounding Thr-50 may represent a potential recognition site on Grx1to be exploited for subsequent design of more selective Grx1 inhibitors. *In silico* tools could also be used to optimize J02 derivatives, as recently employed to identify acrylamide-containing compounds as potential inhibitors for bacterial Grx1 [20].

### Further J02 optimization for interacting with Grx1

We show that J02 is able to interact with Grx1 *in vivo* and *in vitro*. J02 appears to alter antibody binding to Grx1, which may be indicative of structural alterations that expose the antibody-binding epitope(s). Our *in silico* data also indicate that J02 may exert specificity for Grx1 *via* binding to the glutathionyl binding cleft. However, Grx1 clearly is not the only target of J02 intracellularly according to our proteomic survey (supporting data, **S1 Table**) Therefore, improving the selectivity of J02 analogs for Grx1 *in vivo* is necessary to develop a more effective tool for probing the functions of Grx1 in various contexts. As Cys22 of Grx1 is a strong nucleophile, decreasing J02 reactivity by replacing the chlorine moiety with a less reactive halogen, such as fluorine, may target J02 to strong nucleophiles only, thus decreasing off-target effects. Consistent with this concept, a recent study demonstrated enhanced reactivity of the fluoroacetamido moiety when situated in proximity to a thiol [21]. Accordingly, by optimizing the non-covalent binding of J02 analogs in the proximity of the active site Cys-22, the chloroacetamido moiety of J02 could be replaced by the less reactive fluroacetamido moiety, thereby greatly limiting its general reactivity with less reactive protein thiols. In addition, *in silico* tools could also be used to optimize derivative structures of J02 for non-covalent interaction with Grx1, as recently employed for optimizing acetonitrile-derived inhibitors for bacterial Grx1 [20]

### Glutaredoxin as a therapeutic target in inflammatory diseases

Grx1 knockdown has been shown to decrease inflammatory activation of microglia [6], Mueller glial cells [5], and alveolar macrophages [4]. In the present study, we found Grx1 knockdown to inhibit LPS induction of *il6* (**Fig 4**), consistent with Grx1 as a target for anti-inflammatory treatments. *Grx1*^-/-^ mice [22] are viable and appear to have a normal lifespan. However, Grx1 diminution has been shown to contribute to apoptosis in some cell types, such as neurons [23, 24], retinal pigment epithelial cells [25], cardiomyocytes [26] and lung epithelial cells [27]. Therefore, cell-specific delivery of a Grx1 inhibitor would be desirable in order to control potential off-target effects. Furthermore, there are exceptions to the pro-inflammatory role of Grx1in immune cells. Recently, BALB/C^*Grx1*-/-^ mice, lacking Grx1, were shown to mount an enhanced inflammatory response when challenged with house dust mites [28], suggesting that the nature of the Grx1 effect on inflammatory responses may be context- and/or mouse strain-dependent. In addition, overexpression of Grx1 was reported to be anti-inflammatory in leukocytes under particular conditions. Thus, upregulation of Grx1 prevented accumulation of protein-SSG in the monocytes of mice treated with low-density lipoprotein (LDL) plus high glucose; and inhibited monocyte chemoattractant protein-1 (MCP-1)-induced monocyte chemotaxis in the mice [29].

The absence of Grx1 (e.g., *Grx1*^-/-^ mice) has been associated with variable anti-inflammatory effects [7, 10], suggesting that Grx1 may govern a specific inflammatory signature. We found *Grx1* knockdown to prevent *il6* induction, but not that of *tnfa* or *il1b* (**Fig 4**). These results are also consistent with our previous report of *Grx1* knockdown in BV2 cells decreasing LPS-stimulated secretion of IL-6 but not TNF-α [6]. Thus, further development of a selective Grx1 inhibitor could be valuable in the quest for selective agents that would inactivate IL-6 mediated inflammatory responses. Interestingly, C57BL/6^*Grx1*-/-^ mice displayed decreased levels of both *tnfa* and *il6* compared to WT controls [6], suggesting that *Grx1* knockdown in the microglia versus whole brain differentially regulates *tnfa* expression. Further investigation is needed to elucidate the main glutathionylated targets regulated by Grx1 in the microglia, which may explain apparent specificity for Grx1 regulation of *il6* induction.

Covalent compounds are often avoided in the clinic due to concerns of potential side effects following permanent protein adduction. The pharmacodynamics of covalent compounds depends on protein half-life (reviewed in [30]). As Grx1 has been estimated to have a fairly long half-life of ~1.5 days [31], J02 or other Grx1 inhibitors could be administered less frequently, decreasing potential side effects. Moreover, protein stabilization following covalent adduction has been shown to increase half-life in some cases [32], potentially allowing for further reduction in dosage frequency, thus making J02, and other covalent Grx1 inhibitors, attractive therapeutics.

### Anti-inflammatory targets in the J02 interactome

523 proteins were found to be adducted to some extent by J02 (supplemental **S1 Table**). This broad proteomic survey does not provide a quantitative estimate of the extent of adduction, nor can it distinguish whether such adduction is associated with a functional change. As a thiol-selective electrophilic agent, it is expected that J02 would target proteins with sensitive sulfhydryl residues. Indeed the majority of the proteins adducted by J02 have been identified previously in protein databases to be S-nitrosylated (http://dbSNO.mbc.nctu.edu.tw) and/or S-glutathionylated (http://csb.cse.yzu.edu.tw/dbGSH/). As our study was focused on microglia and the observed anti-inflammatory effects of J02, we searched the adducted proteins for those relevant to pro-inflammatory signaling. In this way, the proteomic analysis provided corroborating data, identifying J02 labeling of 4 proteins within the NFκB pathway (p100, p105/p50, inhibitor of nuclear factor kappa-B kinase subunit beta (IKK_β_), and myeloid differentiation primary response gene 88 (MyD88), **Fig 5A**). In addition, we documented J02 binding to p65 *via* immunoprecipitation and western blotting (**Fig 5B**); however, p65 *per se* did not appear in the mass spectrometric analysis. It is possible that p65 was bound to an interactor, such as p50, which could account for the multiple molecular weight bands we observed in the immunoblot with anti-p65. If so, J02 might influence binding partners within the NFκB pathway. As expected, we did observe J02 adducted to Grx1 in both the mass spectrometric and immunoprecipitation-immunoblot experiments, further documenting (i) that J02 interacts with and inhibits Grx1 in the cell, and (ii) that J02 may disrupt the NFκB pathway *via* Grx1 inhibition, which has been shown to decrease NFκB signaling [4, 5]. Therefore, J02 may act as an anti-inflammatory agent in a synergistic manner by negatively regulating the pro-inflammatory NFκB pathway through direct adduction onto member proteins, and by inhibition of the pro-inflammatory action of Grx1.

In summary, this study identifies for the first time a covalent modifier for Grx1 that is effective intracellulary and displays anti-inflammatory efficacy. The covalent mode of action and the presence of the alkyne group on the J02 molecule are properties that make it a promising lead compound whose derivatives would be easy to screen for selectivity and intracellular potency in future studies.

## Materials and methods

### Stepwise synthesis of J02

**Fig 6:**
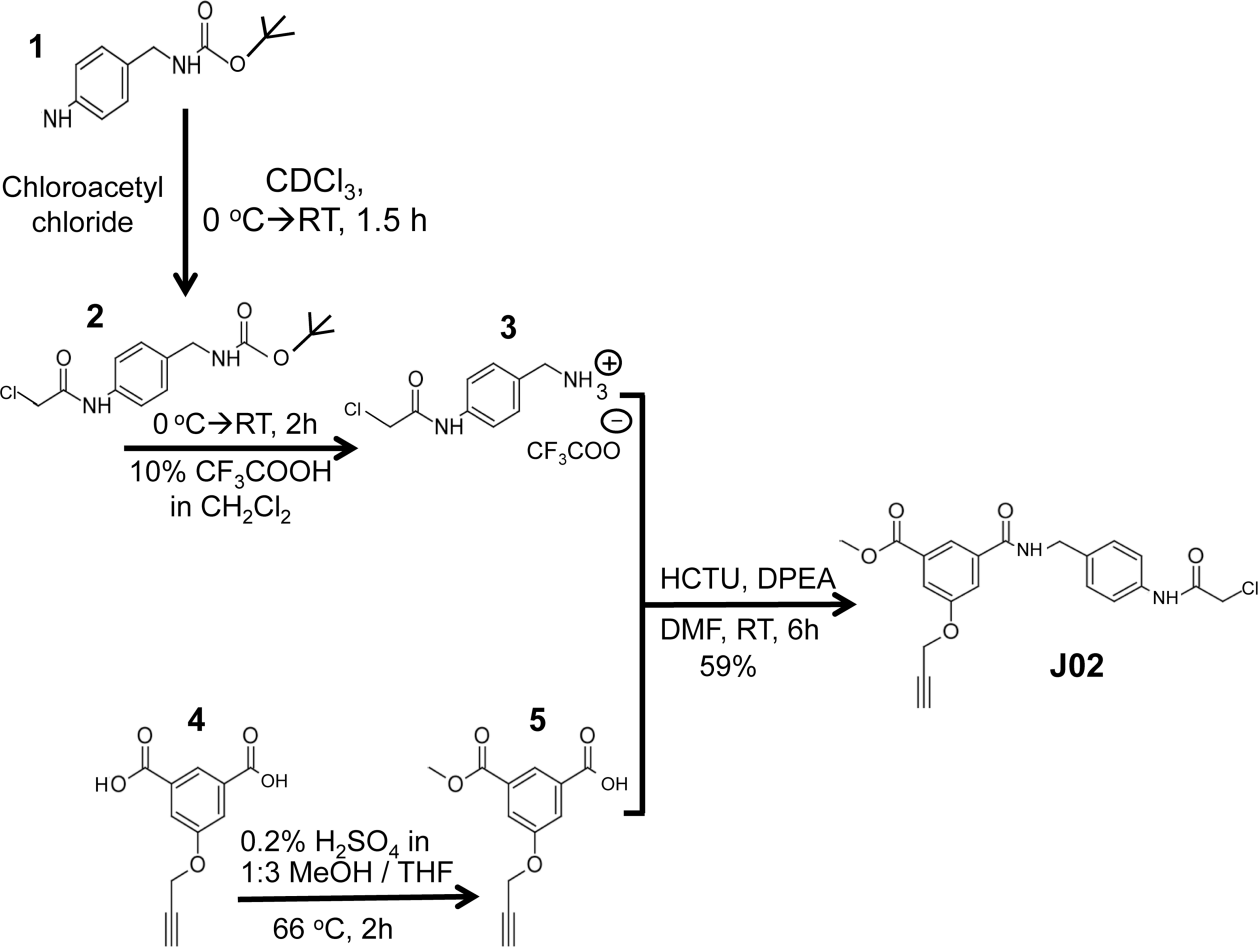
Multistep Synthesis of CWR-J02. This diagram displays the starting compound and illustrates the step-by-step synthesis of J02 involving the preparation, purification, and documentation of purity of the intermediates and the final product. Details are provided under Materials and Methods.

J02, whose structure is shown in **Fig 1A**, was synthesized in a multistep procedure illustrated in **Fig 6**.

#### Synthesis of compound 2

To an ice-cold solution of **1** (150 mg, 0.67 mmol) in deuterated-chloroform (CDCl_3_, 3 ml), chloroacetyl chloride (65 μl, 1.2 eqiuv, 0.81 mmol) was added. To this cold solution triethylamine (141 μl, 1.02 mmol) was added and stirred at RT for 1.5 h. Complete consumption of **1** was observed using TLC analysis (TLC stained with ninhydrin) and proton nuclear magnetic resonance (^1^H NMR) spectroscopy. The volatiles were evaporated under reduced pressure to dryness to obtain crude mixture, **2** as a white solid in 91% (181 mg) yield; R_*f*_ = 0.7 (50% EtOAc/hexane). ^1^H NMR (500 MHz, Chlorofom-*d*) δ 8.42 (s, 1H), 7.54 (d, *J* = 8.3 Hz, 2H), 7.28 (d, *J* = 7.1 Hz, 2H), 4.89 (s, 1H), 4.30 (d, *J* = 4.7 Hz, 2H), 4.21 (s, 2H), 1.47 (s, 9H). This crude solid (160 mg, 0.67 mmol) was re-dissolved in dichloromethane (6 ml) and placed in an ice bath. To this cold solution, trifluoroacetic acid (0.6 ml) was added and stirred at RT for 2 h. Complete consumption of **2** was observed using TLC analysis. The volatiles were evaporated to dryness to obtain a white solid, and this was used in further reactions without purification. ^1^H NMR (500 MHz, Chloroform-*d*) δ 7.69 (d, *J* = 8.5 Hz, 2H), 7.45 (d, *J* = 8.4 Hz, 2H), 4.21 (s, 2H), 4.10 (s, 2H). HRMS (ESI +ve-TOF) for (Free amine) [C9H11N2OCl+Na^+^] calcd., 198.0560, found 198.0556. (Full NMR spectra for each of the intermediates and J02 are shown in supplemental **S1 Fig.**

#### Synthesis of compound 5

Compound **4** was synthesized as reported in [34]. To a solution of **4** (100 mg, 0.33 mmol) in 1:3 methanol: tetrahydrofuran (24 ml), concentrated sulfuric acid (50 μl) was added at RT and heated at 66°C for 2 h under stirring. Partial consumption of **4** was observed using TLC analysis after 2h. Then, all the volatiles were evaporated under reduced pressure to obtain crude mixture, and this was purified by silica gel column chromatography (40–60% EtOAc in Hexane) to obtain ***5*** in 47% (48mg) yield as a white solid (41% of unreacted starting material **4** was recovered, 41 mg); R_*f*_ = 0.4 (75% EtOAc/hexane). ^1^H NMR (500 MHz, Chloroform-*d*) δ 8.42 (s, 1H), 7.90 (s, 2H), 4.83 (d, *J* = 2.1 Hz, 2H), 3.98 (s, 3H), 2.59 (s, 1H). HRMS (ESI +ve-TOF) for [C12H11O5+H^+^] calcd., 235.0606, found 235.0601.

#### Synthesis of J02

To a solution of **3** (35 mg, 0.12 mmol) in dry *N*,*N*-dimethylformamide (DMF, 2ml), compound **5** (28 mg, 1.0 eqiuv, 0.12 mmol) and *O*-(6-Chlorobenzotriazol-1-yl)-*N*,*N*,*N*′,*N*′-tetramethyluronium hexafluorophosphate (HCTU, 73 mg, 1.5 equiv) were added at RT under argon atmosphere. To this solution DIPEA (*N*,*N*-Diisopropylethylamine, 84 μl, 4.0 equiv) was added, and the reaction mixture was stirred at RT for 5 h. Complete consumption of **5** was observed using TLC analysis and the reaction mixture was diluted with water, then extracted using ethyl acetate (EtOAc, 3 X 5ml). The combined organic layer was washed with brine and dried over sodium sulfate (Na_2_SO_4_). The solution was evaporated to dryness to obtain crude mixture, and this was purified by silica gel column chromatography (30–50% EtOAc in Hexane) to obtain **J02** in 59% (29 mg) yield as a white solid; R_*f*_ = 0.6 (50% EtOAc/hexane). ^1^H NMR (500 MHz, DMSO-*d*_6_) δ 10.28 (s, 1H), 9.23 (t, *J* = 5.7 Hz, 1H), 8.14 (s, 1H), 7.77 (s, 1H), 7.67 (s, 1H), 7.55 (d, *J* = 8.4 Hz, 2H), 7.29 (d, *J* = 8.3 Hz, 2H), 4.95 (s, 2H), 4.45 (d, *J* = 5.7 Hz, 2H), 4.24 (s, 2H), 3.89 (s, 3H), 3.64 (s, 1H).^13^C NMR (126 MHz, DMSO-*d*_6_) δ 166.0, 165.2, 165.0, 157.8, 137.7, 136.7, 135.3, 131.6, 128.4, 121.4, 119.8, 119.3, 118.0, 79.3, 79.1, 56.5, 53.0, 44.0, 42.8. HRMS (ESI +ve-TOF) for [C21H19N2O5Cl+Na^+^] calcd., 437.0880, found 437.0883.

### Culture of BV2 cells and treatments

BV2 murine microglial cells were a kind gift of Dr. Gary Landreth (CWRU Department of Neuroscience). As described [6], these BV2 cells were documented previously to respond analogously to primary mouse and human microglia, and they were cultured as described in [6]. The BV2 cells were not used past passage 30. For treatments with J02 or gliotoxin (Sigma, St. Louis, MO), cells were plated at ~2x105 cells/well in 6-well plates in serum-free low glucose Dulbecco’s minimum essential medium (DMEM) (Life technologies, Carlsbad, CA) and let adhere overnight. Cells were treated with indicated concentrations of J02 or equivalent volume dimethyl sulfoxide (DMSO) for 30 min in serum-free low glucose DMEM, media changed, and cells were let recover in media for an additional 60 min. For indicated experiments, cells were stimulated with 1 μg/ml LPS (0111:B4 strain, Sigma, St. Louis, MO) during recovery time.

### Grx1 spectrophotometric assay

Grx1 was purified as previously described [23]. Assay was performed as previously described [35]. Briefly, 0.2 mM nicotinamide adenine dinucleotide phosphate (NADPH) (Roche, Basel, Switzerland) in 0.1 M NaKPO_4_ buffer pH 7.4, 0.5 mM GSH (Sigma, St. Louis, MO), and 2 units/ml glutathione reductase (Sigma, St. Louis, MO) were incubated for 5 min at 30°C with known amounts of purified Grx1 or cell lysates. Reaction was initiated with 0.1 mM cysteinyl glutathione disulfide (Toronto Research Chemicals, Toronto, ON), and decrease in absorbance at 340 nm was read for 5 min. Solution in the absence of Grx1 was used as a blank. Slope of the reaction minus the slope of the no-Grx1 control reaction was used to calculate Grx1 activity. Grx1 activity was normalized to sample protein concentration to calculate Grx1 specific activity.

#### Inhibitor screening

For rapid-throughput screening of potential inhibitors, Grx1 in the standard spectrophotometric assay mixture [35] was pre-incubated for 30 min at 30°C with 40 μM or 120 μM of each of the thiol-reactive compounds, which were added using the automated JANUS pin transfer apparatus (Perkin Elmer, Waltham, MA). Cysteinyl glutathione disulfide was then added to initiate the catalytic reaction, and absorbance at 340 nm was read every 4 min using EnSpire (Perkin Elmer, Waltham, MA). Diminution of the rate of change in A340 nm was ascribed to Grx1 inhibition.

#### Concentration and time dependence studies

For assays building the initial J02 concentration-effect curve (**Fig 1B**), Grx1 in complete assay mix was incubated with indicated concentrations of J02 or equivalent volume of DMSO for 30 min at 30°C, reaction initiated with cysteinyl glutathione disulfide and read as described above. Wells containing J02 or DMSO but lacking Grx1 were used as controls. For the time- and concentration-dependent inhibition (**Fig 1D**), 40 milliunits of Grx1 were incubated with indicated concentrations of J02 in 0.33 M sodium potassium phosphate buffer pH 7.4 at 30°C for indicated time. Mixture was then diluted 20-fold into complete assay mix, and standard spectrophotometric assay was performed. For the modified Kitz-Wilson plot (**Fig 1E**), performed in a fashion analogous to our previous report [13], 6 milliunits of Grx1 and indicated concentrations of J02 were pre-incubated at 30°C for 5 min in 0.33 M sodium potassium phosphate buffer pH 7.4, and then diluted 3-fold into complete assay mix. Grx1 activity was measured in the standard spectrophotometric assay described above.

### Glutathione disulfide reductase (GR) activity assay

Glutathione disulfide reductase (GR) activity was measured as previously described [23]. For J02 inactivation studies, GR was pre-incubated with indicated concentrations of J02 for 30 min prior to activity measurements.

### Mass spectrometric analysis of J02-adducted Grx1

Equimolar concentrations of J02 and Grx1 were incubated in 0.33M sodium potassium phosphate buffer for 30 min at 30°C. ~95% inhibition of Grx1 activity was confirmed using the spectrophotometric Grx1 assay. Samples were then treated with 5x concentration of IAM to block free cysteine residues.

For the protein digestion, the bands were cut to minimize excess polyacrylamide, divided into a number of smaller pieces. The gel pieces washed with water and dehydrated in acetonitrile. The bands were then reduced with dithiothreitol (DTT) and alkylated with iodoacetamide (IAM) prior to the in-gel digestion. All bands were digested in-gel using trypsin by adding 5 μl 10 ng/μl trypsin in 50 mM ammonium bicarbonate and incubating overnight digestion at room temperature to achieve complete digestion. The peptides that were formed were extracted from the polyacrylamide in two aliquots of 30 μl 50% acetonitrile with 5% formic acid. These extracts were combined and evaporated to <10 μl in Speedvac and then resuspended in 1% acetic acid to make up a final volume of ~30 μl for LC-MS analysis.

Samples were analyzed by a Finnigan LTQ-Obitrap Elite hybrid mass spectrometer using Dionex 15 cm x 75 μm id Acclaim Pepmap C18, 2 μm, 100 Å reversed-phase capillary chromatography column for peptides separation. Five μl volumes of the extract were injected and the peptides eluted from the column by an acetonitrile/0.1% formic acid gradient at a flow rate of 0.25 μl/min were introduced into the source of the mass spectrometer on-line. The microelectrospray ion source is operated at 2.5 kV. The digest was analyzed using the data dependent multitask capability of the instrument acquiring full scan mass spectra to determine peptide molecular weights and product ion spectra to determine amino acid sequence in successive instrument scans. The data was searched specifically against the UniProtKB database with Mascot [36] and more specifically against the sequence of Grx1 using Sequest.

### Immunoblotting

Cells were collected and lysed in lysis buffer (10 mM KH_2_PO_4_, 0.1% Triton-X) using freeze-thaw. 30 μg of lysates were solubilized 1:1 in Laemmli sample buffer, reduced with DTT (20–50mM), boiled at 90°C for 10 min, and alkylated with 3-fold excess IAM. Samples were separated on a 4–15% gel (Bio-Rad, Hercules, CA) and transferred to a PVDF membrane. The membrane was blocked in 5% non-fat dry milk in Tris buffered saline 0.05% Tween-20 (TBST) for one hour at room temperature. Membranes were then incubated with either anti-Grx1 antibody (GenScript, Piscataway, NJ) at 1:500 or anti-β-actin (Sigma, St. Louis, MO) at 1:5000 overnight at 4°C. Membranes were washed and incubated with alkaline phosphate conjugated secondary antibodies for one hour. Immunoblots were developed using 7-hydroxy-9H-(1,3-dichloro-9,9-dimethylacridin-2-one) (DDAO) phosphate (Life Technologies, Carlsbad, CA), visualized using Typhoon imager (GE Healthcare, Little Chalfont, United Kingdom), and band intensity was quantified using ImageQuant software.

### RT-qPCR

RT-qPCR was performed as described in [6]. Commercial probes were used for *glrx1* (Mm00728386_s1), *il6* (Mm00434228_m1), *il1b* (Mm00434228_m1) and *tnfa* (Mm00443258_m1) (Life Technologies, Carlsbad, CA). *gapdh* (Mm99999915_g1) was used as a loading control.

### RNA silencing

siRNA was used as previously described [6].

### ATP content assay

Intracellular ATP levels were measured using CellTiter Glo (Promega, Madison, WI) according to manufacturer’s instructions. ATP content was normalized to DMSO treated cells (final concentration 0.4%). Staurosporine (20 μM for 24 hours) was used as a positive control.

### Molecular dynamics simulation

The starting coordinates for Grx1 were obtained from the NMR structure (PDB ID: 1JHB [37]). J02 was positioned in relation to Grx1 with the chloroacetamido group of J02 near the active-site Cys-22 of Grx1, based on experimental data from Mass spectrometry. The Autodock Vina plugin in UCSF Chimera [38] was used to produce different starting orientations of J02 near the predicted active site of Grx1. This program treated Grx1 as a rigid receptor and required a rectangular search space, which was assigned based on the predicted binding site near Cys-22. Six possible orientations of J02 were found and the lowest energy possibility was selected. This complex provided the starting coordinates for molecular dynamics calculations using Assisted Model Building with Energy Restraints (AMBER) [39] with the General Amber Force Field (GAFF) [39]. The complex was prepared for molecular dynamics simulations using the LEaP module of AMBER, which included adding sodium ions (0.15 M) to mimic physiological conditions. The unit cell was configured with a box that extended 10 Å beyond the protein/inhibitor complex resulting in a box size of 67 x 76 x 73 Å. The complex was then solvated with TIP3P water molecules. Nanoscale molecular dynamics (NAMD) [40] simulations were run on the Ohio Supercomputer Center [41] using 420 processors and two graphics processing units (GPUs). Each system was equilibrated to 300K and minimized for 20 ns before beginning a 50 ns molecular dynamics simulation.

The results of the molecular dynamics simulations were analyzed using the NAMD energy plugin in Virtual Molecular Dynamics (VMD) [42]. NAMD energy was used to calculate the non-bonded interaction energy between each residue of Grx1 and J02 both before and after the 50 ns molecular dynamics simulation.

### Proteome mass spectrometry analysis

#### Preparation of BV2 cell lysates

BV2 microglial murine cells cultured in low glucose (5 mM) DMEM with 10% FBS were serum starved overnight. Cells were then treated +/- J02 (40 μM) for 30 minutes in serum free, low glucose DMEM. The media was then changed and the cells were allowed to recover for one hour. The cells were then harvested and the pellets washed with phosphate buffered saline (PBS). After washing, the pellets were resuspended in an appropriate amount of PBS and then sonicated with an ultrasonic tip sonicator (Cole Parmer, Vernon Hills, IL). The lysates were separated by centrifugation at 45,000 rpm for 45 minutes at 4°C to obtain soluble and insoluble lysate fractions. The soluble fraction was collected and the pellet discarded. Protein concentrations were determined using the DC Protein Assay kit (Bio-Rad, Hercules, CA).Eight confluent 150 mm plates of BV2 cells per sample were treated with 40 μM J02 or DMSO for 30 min, media changed, and cells were left to recover for 60 min. Resulting pellets were prepared for tandem mass spectrometry as described in [43].

#### Click chemistry and streptavidin enrichment of J02 labeled proteins

J02 labeled BV2 cell lysates (500 μl, 2 mg ml^-1^) in PBS were subjected to click chemistry. Biotin azide (200 μM from 100x stock in DMSO), TCEP (1 mM, 50x stock in water), TBTA (100 μM, 17x stock in tBuOH:DMSO 4:1), and copper (II) sulfate (1 mM, 50x stock in water) were added to the cell lysate. Samples were incubated at room temperature for 1 hour to allow for the cycloaddition reaction to occur. Samples were then centrifuged for 10 minutes at 4°C to pellet the precipitated proteins. Protein pellets were then resuspended in cold methanol by tip sonication followed by centrifugation. Following a second methanol wash, pelleted proteins were solubilized in a 1.2% SDS/PBS solution via tip sonication and incubation at 85°C for 5 minutes. Samples were then diluted with 5 ml PBS to lower the concentration of SDS to 0.2%. Next, samples were incubated with 100 μl streptavidin agarose beads (Thermo Fisher Scientific, Waltham, MA) at 4°C for 16 hours. Samples were then washed with 0.2% SDS/PBS (5 ml), PBS (3 x 5 ml), and water (3 x 5 ml). The streptavidin agarose beads were pelleted between each wash step by centrifugation (1,400 g, 3 min).

#### On-bead trypsin digestion

The beads were suspended in a solution of 6 M Urea/PBS (500 μl) and 10 mM dithiothreitol (DTT, 20x stock in water), followed by incubation at 65°C for 20 minutes. Next, iodoacetamide (20 mM, from 50x stock in water) was added to each sample and incubated at room temperature for 30 minutes. The beads were pelleted (1,400 g, 3 minutes) and resuspended in 200 μl of 2 M Urea/PBS, 1 mM CaCl_2_ (100x stock in water), and 2 μg trypsin (Promega, Madison, WI). On-bead trypsin digestion was allowed to proceed overnight at 37°C with agitation. The beads were pelleted (1,400 g, 3 min) and the supernatant collected. The beads were washed with water (2 x 50 μl) and the washes were combined with the supernatant. Formic acid (15 μl, Thermo Fisher Scientific, Waltham, MA) was then added to each sample and the samples were stored at -20°C until mass spectrometry analysis.

#### Tandem liquid chromatography

Mass spectrometry (LC/LC-MS/MS) analysis—LC/LC-MS/MS analysis was performed on an LTQ Orbitrap Discovery mass spectrometer (Thermo Fisher Scientific, Waltham, MA) coupled to an Agilent 1200 series HPLC (Agilent Technologies, Santa Clara, CA). Tryptic digests were pressure loaded onto a 250 μm fused silica desalting column packed with 4 cm of Aqua C18 reversed phase resin (Phenomenex, Torrance, CA). Peptides were then eluted onto a biphasic 100 μm fused silica column with a 5 μm tip, packed with 10 cm of C18 and 4 cm of Partisphere SCX (Whatman, Pittsburgh, PA). Elution of the peptides from the desalting column into the biphasic column occurred using a gradient of 5–100% Buffer B in Buffer A (Buffer A: 95% water, 5% acetonitrile, 0.1% formic acid; Buffer B: 20% water, 80% acetonitrile, 0.1% formic acid). The peptides were eluted from the SCX onto the C18 resin and then into the mass spectrometer using the four salt steps outlined in [43]. The flow rate of buffer through the fused silica column was set to 0.25 μl min^-1^ and the spray voltage was set 2.75 kV. One full MS scan (400–1800 MW) was followed by 8 data dependent scans of the n^th^ most intense ions with dynamic exclusion enabled.

#### Mass spectrometry data analysis: +/- J02

Two biological replicates each of J02 (40 μM) or DMSO treated BV2 cells were subjected to LC/LC-MS/MS analysis as outlined above. The generated tandem MS data was searched using the SEQUEST algorithm against the mouse UniProt database. A static modification of +57.0215 Da on cysteine was added to account for alkylation of cysteine residues with iodoacetamide. The SEQUEST output files were then filtered using DTASelect v2.0 to generate a list of proteins identified with a false-discovery rate of < 5%. The resulting peptides were then further filtered to display proteins identified in J02 treated samples with an average of 10 spectral counts or greater across the biological replicates. For each of these proteins, the fold-change in spectral counts between J02 treated samples and DMSO samples was calculated and the data are listed sequentially starting with those proteins displaying the highest fold-change in spectral counts in the J02 treated samples relative to the DMSO treated samples (see supplemental **S1 Table**).

### Statistical analysis

Differences between measurements were analyzed using GraphPad Prism using 2-tailed Student’s t-test with Welch’s correction; p-values less than 0.05 are considered significant.

## Supporting information

S1 Table This supplementary table provides the list of proteins that were identified to be adducted by J02 to a detectable extent by mass spectrometry. The proteins are listed sequentially starting with those proteins displaying the highest fold-change in spectral counts in the J02 treated samples relative to the DMSO treated samples.
(XLSX)

S1 Fig This supplementary figure provides the actual ^1^H NMR spectra for the J02 compound and its synthetic precursors. Descriptions of the NMR spectra are provided under Materials and Methods in the main text where the synthetic scheme (**Fig 6**) is described.
(DOCX)

S2 Fig This supplementary figure provides the uncropped blots for Figs 3D and 5B, showing where the blots were cropped. (DOCX)

S3 Fig This supplementary figure provides the data showing that cytosolic Trx1 in BV2 cells is not inhibited by 40 μM J02, the concentration which inhibits Grx1 in the cells by 50%. (DOCX)
