# Supplementary material for: Novel chloroacetamido compound CWR-J02 is an anti-inflammatory glutaredoxin-1 inhibitor
Source: PLoS One. 2017 Nov 20;12(11):e0187991. doi: 10.1371/journal.pone.0187991 (PMC5695812; doi:10.1371/journal.pone.0187991)
Supplement: S1 Fig — Descriptions of the NMR spectra are provided under Materials and Methods in the main text where the synthetic scheme (Fig 6) is described. (DOCX) [file pone.0187991.s002.docx]

**S1 Fig.**  NMR Spectra of J02 and synthetic precursors

1. ^1^H NMR of **CWR-J02**:

1. 13C NMR of **CWR-J02**:

1. ^1^H NMR of **Compound** **2**:

1. ^1^H NMR of **Compound 3**:

1. ^1^H NMR of **Compound 5**:
