# Supplementary figures and images for: Novel chloroacetamido compound CWR-J02 is an anti-inflammatory glutaredoxin-1 inhibitor
Source: PLoS One. 2017 Nov 20;12(11):e0187991. doi: 10.1371/journal.pone.0187991 (PMC5695812; doi:10.1371/journal.pone.0187991)

**S2 Fig.** Uncropped Western Blots


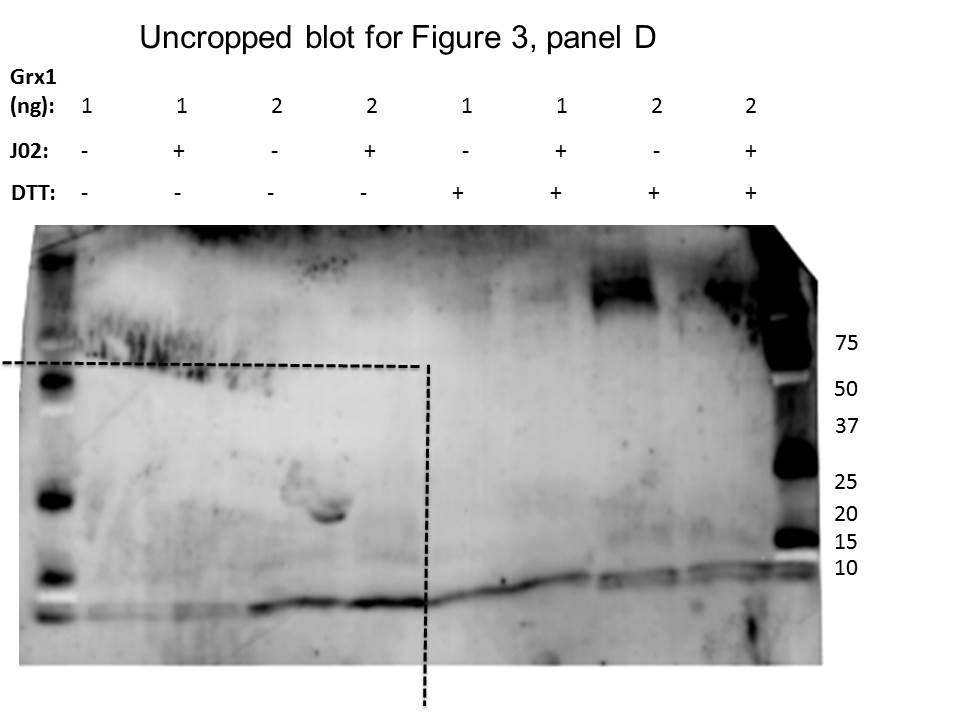


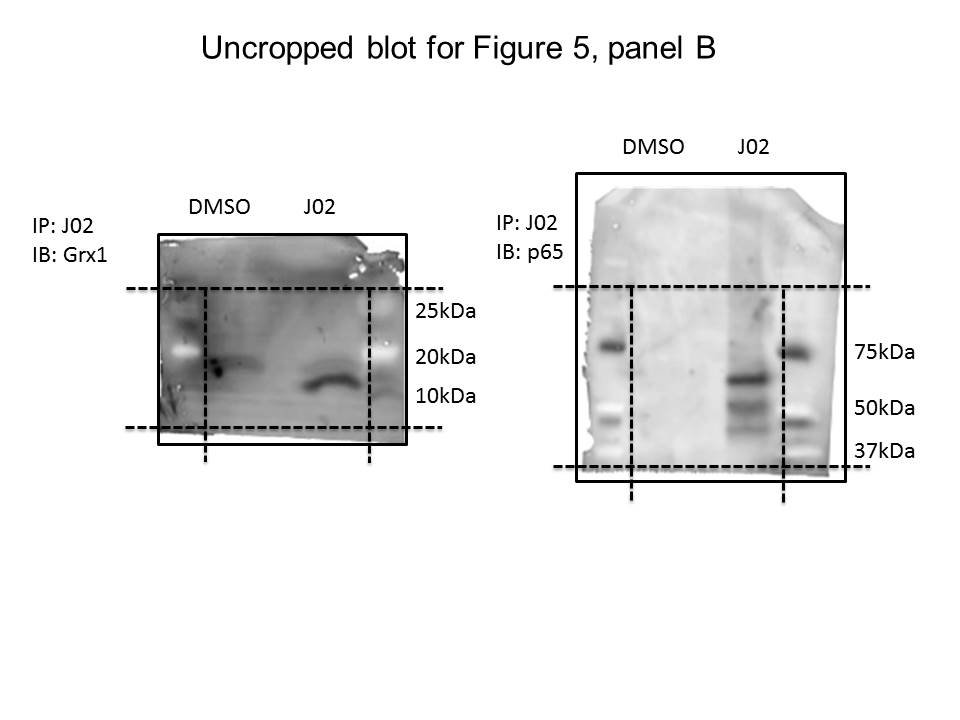

Supplement: S2 Fig — (DOCX) [file pone.0187991.s003.docx]
