# Supplementary material for: Novel chloroacetamido compound CWR-J02 is an anti-inflammatory glutaredoxin-1 inhibitor
Source: PLoS One. 2017 Nov 20;12(11):e0187991. doi: 10.1371/journal.pone.0187991 (PMC5695812; doi:10.1371/journal.pone.0187991)
Supplement: S3 Fig — (DOCX) [file pone.0187991.s004.docx]

**S3 Fig.** Thioredoxin Activity in Microglia Treated with J02


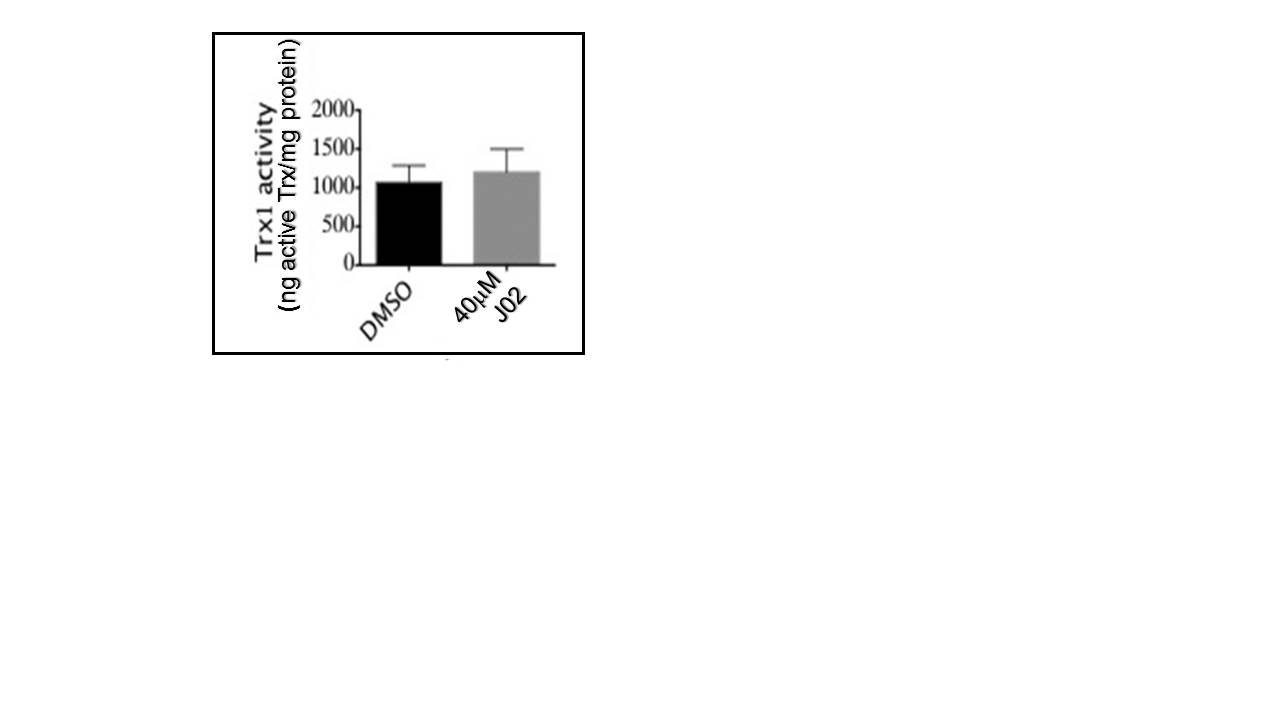


Trx1 activity in model microglia (BV2 cells) treated with 40 mM J02 for 30 min, medium changed, and left to recover for 1 hr. The amount of catalytically active Trx1 was measured in lysates of the BV2 cells by means of the IMCO (Sweden) kit, following manufacturer’s instructions (<https://www.caymanchem.com/pdfs/11527.pdf>). The assay monitors Trx-dependent reduction of fluorescently-labeled insulin, coupled to NADPH and Trx reductase (TR) which are in excess.
